# Targeted Exon Capture and Sequencing in Sporadic Amyotrophic Lateral Sclerosis

**DOI:** 10.1371/journal.pgen.1004704

**Published:** 2014-10-09

**Authors:** Julien Couthouis, Alya R. Raphael, Roxana Daneshjou, Aaron D. Gitler

**Affiliations:** Department of Genetics, Stanford University School of Medicine, Stanford, California, United States of America; Georgia Institute of Technology, United States of America

## Abstract

Amyotrophic lateral sclerosis (ALS) is a devastating neurodegenerative disease that results in progressive degeneration of motor neurons, ultimately leading to paralysis and death. Approximately 10% of ALS cases are familial, with the remaining 90% of cases being sporadic. Genetic studies in familial cases of ALS have been extremely informative in determining the causative mutations behind ALS, especially as the same mutations identified in familial ALS can also cause sporadic disease. However, the cause of ALS in approximately 30% of familial cases and in the majority of sporadic cases remains unknown. Sporadic ALS cases represent an underutilized resource for genetic information about ALS; therefore, we undertook a targeted sequencing approach of 169 known and candidate ALS disease genes in 242 sporadic ALS cases and 129 matched controls to try to identify novel variants linked to ALS. We found a significant enrichment in novel and rare variants in cases versus controls, indicating that we are likely identifying disease associated mutations. This study highlights the utility of next generation sequencing techniques combined with functional studies and rare variant analysis tools to provide insight into the genetic etiology of a heterogeneous sporadic disease.

## Author Summary

Amyotrophic lateral sclerosis (ALS), also known as Charcot disease or Lou Gehrig's disease, is one of the most common neuromuscular diseases worldwide. This disease is characterized by a progressive degeneration of motor neurons, leading to patient death within a few years after onset. Despite the fact that most ALS cases are sporadic, most of the ALS genetic studies have focused on familial forms, leading to the genetic determination of cause for 70% of cases of familial ALS but for only 10% of sporadic ALS cases. This, coupled with the dearth of families available for study, suggests that researchers should begin tapping into the relatively untouched reservoir of available sporadic samples to identify novel genetic causes of sporadic ALS. Here we take advantage of high-throughput target sequencing techniques to test four different hypotheses about the genetic causes of ALS in sporadic ALS and uncover new candidate genes and pathways implicated in ALS.

## Introduction

Amyotrophic lateral sclerosis (ALS) is a neurodegenerative disease that primarily affects motor neurons, resulting in progressive paralysis and death [1]. About 2 in 100,000 people per year are diagnosed with ALS and the disease is often ruthlessly progressive, with death occurring between 2–5 years after disease onset. Currently, there is only one treatment for ALS, riluzole, which extends lifespan by approximately three months. The majority of ALS cases are sporadic, meaning that they occur with no family history of the disease (sALS). The remaining 5–10% of cases are familial (fALS), where the disease is inherited in a Mendelian, generally dominant, fashion within a family. While sALS has a complex etiology, with both environmental and genetic factors thought to play a role, in recent years several genes have been linked to both fALS and sALS.

Previous studies to identify causative genes in ALS have primarily been carried out in families and some genes identified in fALS studies have subsequently been found to be mutated in sALS cases as well (e.g., [2]–[4]). Indeed, in the past few years there has been an explosion in the number of genes linked to ALS, in part due to the accessibility of next generation sequencing techniques [1]. However, as the number of large uncharacterized fALS pedigrees decreases, new approaches will be needed to expand the genetic landscape of ALS. One can imagine several approaches to this problem. First, candidate approaches can be carried out, based on what is currently known about the biology of ALS. For example, we previously used a candidate approach to identify novel ALS disease genes by looking for genes that behaved similarly to the well-known ALS disease genes, *TARDBP* and *FUS*. Both TDP-43 (the protein product of the *TARDBP* gene) and FUS contain RNA Recognition Motifs (RRMs) and prion-like domains, and aggregate and are toxic in yeast, mimicking their behavior in disease [5], [6]. Using these characteristics, we looked for other RRM-containing proteins that also aggregated and were toxic in yeast; of the 133 proteins we tested, 38 aggregated and were toxic in yeast [7]. Out of these we were particularly interested in two genes, *TAF15* and *EWSR1*; first because they also contain prion-like domains and second because they are in the same gene family as *FUS* [7], [8]. Subsequent Sanger sequencing of *EWSR1* and *TAF15* in sALS cases led to the identification of variants that were present in ALS cases but not in controls [7], [8]. Since then some of the other candidate genes from this yeast screen were independently linked to ALS or related neuromuscular disorders. For example, mutations in *hnRNPA1* and *hnRNPA2/B1* were found in ALS families and in multisystem proteinopathy [9], *TIA1* mutations were identified in Welander distal myopathy [10], [11] and also associated with stress granule formation in ALS [12]. Finally a mutation in *hnRPDL* was found to cause limb-girdle muscular dystrophy 1G [13].

Another approach is to take advantage of small families. Variants in *ErbB4* were recently associated with ALS using small pedigrees and sporadic cases [14]. We used a similar approach to identify new candidate disease genes by studying trios – families with an affected proband and two unaffected parents – to ask if we could uncover *de novo* mutations that are present in the affected proband, but not in the unaffected parents. This approach allowed us to identify approximately 25 new candidate ALS disease genes, including *SS18L1/CREST*, which is a member of the nBAF complex and is required for proper neurite outgrowth [15].

Finally, one could also imagine taking advantage of the large number of sALS patient samples, as has been done with the many genome wide association studies (GWAS) previously performed for ALS [16], [17]. While studies performed solely with sALS cases lack the Mendelian power that familial studies have, these studies make up for this deficiency with numbers – there are large numbers of sporadic ALS samples available for study. Unfortunately GWASs are often inconclusive and sometimes the results cannot be replicated [16]–[18]. For ALS, several independent GWASs pinpointed the chromosome 9p21 region as associated with ALS [17]–[20]. As the numbers of cases and controls analyzed by GWAS increases, additional loci are being implicated and warrant further investigation and validation [18], [21].

However, evidence from twin and other studies strongly suggest that there is a heritability component to sALS and thus genetic approaches, such as whole genome or more targeted sequencing methods, should be useful in identifying causative variants in sALS [18], [22]–[24]. Additionally, *de novo* mutations in genes like *SOD1* and *FUS* have been identified in sporadic cases of ALS [25]–[27]. The main challenge of studying sALS is that the variants responsible for ALS are likely not shared between individuals and may not even be in the same gene. The challenge of statistically evaluating rare variants in biologically relevant genes has been addressed by rare variant binning methods [28], [29].

As such, we decided to take advantage of the proliferation and reduction in cost of next generation sequencing techniques to test this idea in the untapped resource of sALS samples. Because GWAS does not pick up the disease relevant rare and novel variation and full exome or genome sequencing analysis would focus on candidate genes anyway, we targeted a large set of candidate disease genes in a cohort of sALS patients and controls [30]. This allowed us to test several hypotheses: (1) can we find new variants in known ALS disease genes? (2) can we find new variants in genes implicated by GWAS and other studies and test previously identified variants in our cohort? (3) can we find new variants or confirm previously identified ones in the set of candidate genes generated from our previous ALS trios study [15]? And (4) can we identify an enrichment in variants in genes containing RNA recognition motifs (RRMs), like *TDP-43* and *FUS* [7], [8]?

We sequenced all exons of 169 genes in 242 ALS patients and 129 age-matched controls from the Coriell Cell Repository (http://ccr.coriell.org/). While no one gene was significantly enriched between cases and controls, we saw an enrichment of deleterious rare and novel variation across previously implicated ALS genes between cases and controls. Additionally, we identified new variants in known ALS disease genes, as well as in genes associated with ALS, found no association between several previously identified SNPs and ALS in our population, with the exception of *APOE*, and identified new variants in genes identified through our trio and RRM studies [7], [8], [15]. This study demonstrates the utility of hypothesis-driven targeted sequencing for identifying disease-relevant novel and rare variation, as we did observe an increase in novel and rare variants in our cases versus controls, but also highlights the challenges and limitations of such studies and the need for further studies with more patient and control samples. Moreover, we also present the application of rare variant binning to show that sALS individuals have a higher burden of deleterious mutations in previously implicated ALS genes.

## Results

### Targeted sequencing of ALS candidate genes

We sequenced 169 candidate genes in 242 patients and 129 age-matched controls using the HaloPlex technology with a custom designed library (Agilent; Table S1). This method results in the exon capture of a specific customized library of genes allowing for the rapid screening of a limited number of genes across many samples. We initially attempted to sequence 276 patients and 184 controls, but the HaloPlex enrichment did not work for all samples. For the samples that were included, on average, 50,000,000 reads with a mean coverage of 55 reads per base and 65% of the bases having more than 10 reads was generated for each sample.

**Figure 1:**
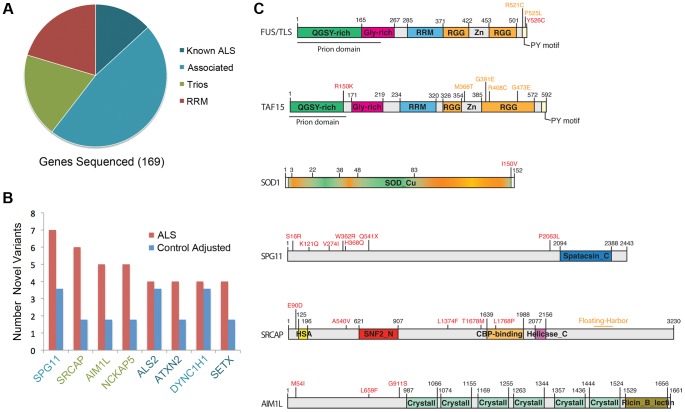
Sequenced genes and hits category repartitions. All genes fall into the four following categories: known ALS disease genes (Known ALS), genes potentially associated with ALS (Associated), candidate genes from a previously published analysis of ALS trios (Trios) and genes containing RNA Recognition Motifs (RRM) bearing high prion scores or are very toxic when expressed in yeast. A) Pie chart showing the four categories of genes sequenced. B) Bar graph showing the number of rare or novel variants found in cases in controls for the genes in which the most variants were found. The number of variants found in controls was adjusted for a control cohort of the same size than the ALS cohort. C) Localization of novel variants (in red) identified in this study for some of the top hits. Position of some selected variants already linked with ALS, or other diseases (Floating-Harbor syndrome, SRCAP), are indicated in orange (or in orange heat map, SOD1).

The pre-determined candidate genes sequenced in this study fell into four categories (Figure 1A; Table S1): genes with prior evidence to affect ALS (Known ALS), genes that were potentially associated with ALS through GWAS (Associated), candidate genes from our previously published analysis of ALS trios (Trios) [15] and genes containing RNA Recognition Motifs (RRM) that also had high prion scores or were very toxic in our previously published yeast screen [31]. Some genes fell into multiple categories; for example, *FUS* is categorized as a known ALS gene, however, it also contains an RRM and prion domain. In total, we found 134 novel variants (not present in ESP6500, 1000genomes, or dbSNPv137) in ALS patients versus 61 novel variants in controls (Tables S2, S3). One variant was found in two ALS patients and in one control and was removed from further analysis, as its presence in both populations suggests that it is likely a benign polymorphism.

Given that rare variants can also contribute to disease [32], we asked how many rare variants were present in our samples (variants that were already present in either ESP6500, 1000genomes or dbSNPv137 but at very low frequencies). We chose a frequency of equal to or less than 0.000538 in the ESP6500 database (http://evs.gs.washington.edu/EVS/) as our cut off point, since this is the frequency of one of the known, disease causing, *SOD1* mutations present in our dataset (p.D91A, rs80265967, [33]). Variants found in both cases and controls were removed, resulting in the identification of 99 rare variants in ALS cases and 41 in controls (Tables S4 and S5).

We then asked how many of our case and control samples harbored novel or rare variants. Of the 242 patients sequenced, we found rare or novel variants in 144 of these, suggesting that at minimum approximately 40% of the cases have no potential genetic cause for ALS that we could identify with our sequencing method and set of candidate genes. We did not expect to be able to explain all ALS cases, as genes outside this library are also likely to be responsible for ALS, noncoding regions are also potentially involved (e.g. regulatory regions or *C9ORF72*-like intronic expansions [20]), and environmental factors are also known to play a role in the etiology of ALS [1]. Of the 129 control samples that were sequenced, novel or rare variants were found in 69 samples (47% without).

While no single gene emerged as significantly enriched in our analysis, we did find several genes that trended towards more novel and rare variants in ALS patient samples versus controls (Figure 1B and Table S1), including *SRCAP* (trios, 10 novel or rare variants in patients versus 2 in controls) and *AIM1L* (trios, 5 novel or rare variants in patients versus 1 in controls). These, and other individual genes, are discussed in more detail below.

### Known ALS disease genes

There are 22 genes that we considered to be ALS disease causing genes (Table S1) and we tested whether we could uncover either known disease causing variants or novel variants in our cohort. Genes that were listed in OMIM (http://www.ncbi.nlm.nih.gov/omim) as causing or increasing susceptibility to ALS were included in this list, as well as two genes that have very recently been tied to sporadic ALS and frontotemporal lobar degeneration (FTLD), *EWSR1* and *TAF15* [7], [8], [34]. This patient set from Coriell has been prescreened for *TARDBP* mutations, and we confirmed that none of the patients carried mutations in this gene. As is perhaps to be expected, we uncovered known ALS causing mutations in *SOD1* in three of our patient samples (Table S4 and S6, Figure 1C); however, these were the only known ALS causing mutations that we uncovered. In addition to these known variants, we found 22 novel variants in known ALS disease genes in cases and 8 novel variants in controls (Table S7). Of particular interest was that several of these novel variants are located very close to known ALS-linked variants, suggesting that they may indeed be causative. For example, we found novel variants in *ANG*, *ALS2*, *ATXN2*, *FUS*, *SETX*, *SOD1*, and *TAF15* (for full list, see Table S7).

#### Frequencies of ALS disease genes

Recent studies have asked to what percentage the known ALS disease genes contribute to familial and sporadic ALS in different populations (e.g. *SOD1*, *TARDBP*, *FUS*, *ANG*) [35]–[38]. We also asked this question of our dataset (with the exception of *TARDBP*, as our cohort lacks mutations in this gene). Of the remaining 21 genes conclusively linked to ALS (Table S1), we found 33 novel or rare variants in 32 patients. Therefore, variants in known ALS disease genes can potentially be causative for disease in 13% of our patient samples. The genes with the highest number of variants were *DCTN1* (n = 5, frequency  = 2%), *SETX* (5, 2%), *SOD1* (4, 1.7%), *ALS2* (3, 1.2%) and *ATXN2* (3, 1.2%; one novel variant was found in both cases and controls and is thus considered not to be causative). We did not find any significant correlation between gene size and number of new variants identified. We found two novel variants each for *FIG4*, *PRPH*, and *TAF15*, for a frequency of 0.8%. We only found one variant each for *ANG*, *EWSR1*, *FGGY*, *FUS*, and *NEFH*, for a frequency of 0.4%.

Given that some recent studies have asked at what frequency a subset of known ALS disease genes are present in certain populations, we could compare the frequencies we obtained from our cohort of North American patients to previously published frequencies from Italian, Irish and Korean cohorts (Table 1) [35]–[38]. Frequency information was available for *ANG*, *FUS*, *OPTN* and *SOD1* in all three of these populations. For these four genes, our population has allele frequencies more similar to the Italian population than to the Korean population. A more extensive comparison of genes was possible with the Irish cohort [35] (Table 2); the frequencies appeared very variable between the published Irish cohort and our North American cohort. However, as described further below, similar mutations and trends were uncovered.

**Table 1:** ALS variants prevalence among different genetic backgrounds.

Gene	American (242)	Irish (444)	Italian (1003)	Korean (258)
ANG	0,41%	0,00%	0,60%	0,00%
FUS	0,41%	0,45%	0,40%	2,71%
OPTN	0,00%	0,23%	0,40%	0,00%
SOD1	1,65%	0,00%	1,99%	3,88%
Total	2,48%	0,68%	3,39%	6,59%

Prevalence of ANG, FUS, OPTN and SOD1 variants in ALS between our American cohort and Irish, Italian and Korean populations [35]–[38].

**Table 2:** Comparison with Irish population.

	Gene	American	Irish
Mendelian	**ALS2**	1,24%	1,58%
	**ANG**	0,41%	0,00%
	**FUS**	0,41%	0,45%
	**OPTN**	0,00%	0,23%
	**SETX**	2,07%	2,48%
	**SOD1**	1,65%	0,00%
	**VAPB**	0,00%	0,00%
	**VCP**	0,00%	0,23%
Low penetrance/Tentative ALS genes	**ATXN2**	1,22%	0,00%
	**CHMP2B**	0,00%	0,45%
	**DCTN1**	2,07%	0,45%
	**DPP6**	1,65%	0,23%
	**ELP3**	0,41%	0,68%
	**FGGY**	0,41%	0,23%
	**FIG4**	0,83%	0,00%
	**GRN**	0,41%	0,00%
	**HFE**	1,65%	0,23%
	**ITPR2**	1,24%	0,23%
	**MAPT**	0,00%	0,45%
	**NEFH**	0,41%	0,00%
	**PON1**	0,00%	0,00%
	**PON2**	0,00%	0,23%
	**PON3**	0,00%	0,00%
	**PRPH**	0,83%	0,00%
	**SIGMAR1**	0,00%	0,00%
	**SPG11**	4,13%	1,58%
	**UNC13A**	1,24%	0,23%

Genes are sorted into two categories, depending on whether they where implicated in the Mendelian form of the disease (equivalent to the “known ALS” category in this paper) or are low penetrance/tentative ALS genes (“associated”), as used in the original publication [35].

#### ALS2 and SETX

Homozygous mutations, generally small deletions leading to frameshifts, in ALS2 have been shown to be causative for juvenile ALS [39], [40], so we were particularly interested to find novel variants in our adult ALS samples. We uncovered two novel variants in ALS2, p.T700A, which we found in two patients, and p.V763I (Table S2). For these three patients with *ALS2* mutations, the age of onset was well past that considered to be juvenile ALS [1]. A recent study also identified point mutations in *ALS2* in adult ALS patients, one of which we also found – leading to the p.T700A variant [35]. This mutation has now been identified in four adult sporadic ALS cases, with 6 other novel or rare variants identified (this study and [35]). Combined, these results suggest that heterozygous point mutations in ALS2 may be causative for adult onset, sporadic ALS.

Mutations in *SETX* have also previously been linked to autosomal dominant juvenile ALS (ALS4) and to autosomal recessive spinocerebellar ataxia 1 (SCAR1) [41], [42]. We found four novel variants in SETX in patient samples (p.Y1681C, p.S269L, p.R2540L, and p.I974T), one of which was nearby a mutation previously linked to SCAR1 (p.M274I, [43]), and one rare variant (p.R1846H) (Tables S2, S4). Novel or rare mutations in *SETX* have also been identified in an adult Chinese patient with ALS [44] and in 11 sporadic or familial cases in an Irish patient cohort [35]; here, we provide further evidence linking *SETX* to adult onset ALS. Similar to ALS2, our findings and others suggest that *SETX* may play a role in adult onset ALS.

#### ATXN2 and C9ORF72

While intermediate length expansions in the polyglutamine (polyQ) tract of ATXN2 were previously shown to be a risk factor for ALS [45], to date no one has asked if point mutations in the *ATXN2* gene are linked to ALS. Through our HaloPlex analysis, we sequenced all exons of *ATXN2* and found three novel variants. Two novel variants were found only in ALS patients (p.S72F and p.S1125C), suggesting that they may be linked to disease, and one was found in two patient samples and in one control (p.P71L). Even if this last one is likely not to be pathogenic, its close proximity to p.S72F could suggest that it may have reduced penetrance.

Hexanucleotide repeat expansions in an intron of *C9ORF72* were recently shown to be causative for ALS [19], [20]; the Coriell repository ALS samples have previously been analyzed for expansions in the intron of C9ORF72 and 11.8% of them carry an expanded allele [46]. Our selected patient cohort only contains 4 samples carrying a *C9ORF72* expansion (1.65%). Since previous studies have found that patients harboring a *C9ORF72* expansion can sometimes also carry a second mutation in a disease-causing gene [47], we asked if this was true of our patient cohort. For only one of our patient samples for which we identified a novel variant was there was also an expansion in *C9ORF72*; this sample carried a novel variant in CDH13 (p.K63N), which is a gene that has been previously studied in ALS [48] (Table S2). We also found a rare variant in a patient with a *C9ORF72* expansion in CNOT1 (Table S4). Similar to *ATXN2*, we set out to determine if we could identify novel or rare variants in the coding sequence of *C9ORF72* and we discovered one rare variant, p.T49R, in an ALS patient.

#### FUS

**Figure 2:**
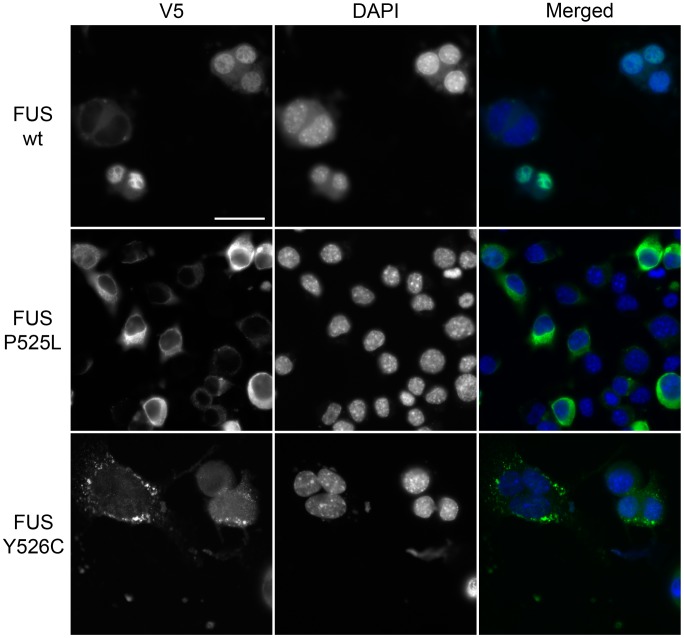
Functional characterization of newly identified p.Y526C FUS variant. V5-tagged wild type FUS, ALS causative P525L and newly identified Y526C variants were transfected into N2A mouse neuroblastoma cells and their localization was determined by fluorescence microscopy. As previously reported wild type FUS localized in the nucleus while P525L and Y526C FUS were mislocalized to the cytoplasm. Scale bar is 30µm.

We found a new variant in FUS, p.Y526C (NM_001170634:c.A1574G), which is present in the proline-tyrosine nuclear localization signal (PY-NLS) of the protein. This NLS is recognized by the nuclear import factor karyopherin β2/transportin. Several other disease-causing mutations are localized in this domain and have been shown to impair transportin-mediated nuclear import [49](Table S7; Figure 1C). We therefore designed a functional assay to test the impact of this new variant on FUS localization. We transfected mouse neuroblastoma (N2A) cells with V5-tagged wild type (WT), P525L or Y526C FUS constructs. WT FUS localized to the nucleus and the P525L mutant protein redistributed to the cytoplasm, consistent with previous reports [50]. The novel Y526C mutant protein also redistributed to the cytoplasm where it was often recruited in small granular accumulations (Figure 2). This confirms that the newly identified Y526C FUS variant does not behave like the wild-type protein, but rather is mislocalized to the cytoplasm like other known disease-causing FUS variants (e.g. p.P525L [49], [51]). Thus, this novel FUS variant is damaging and likely to be causative of ALS.

#### EWSR1 and TAF15

We recently identified a role for *EWSR1* and *TAF15* in ALS by studying candidate genes that were similar to the previously identified ALS disease genes *TARDBP* and *FUS* [7], [8]. All four of these genes encode RNA binding proteins with RNA Recognition Motifs (RRMs) and prion-like domains (discussed in more detail below). Moreover FUS, EWSR1 and TAF15 are members of the same family of proteins (FET) with a highly similar domain organization [52]. Previously, we only looked for mutations in the C-terminal domains of EWSR1 and TAF15, as this is where the majority of FUS mutations had been previously identified. This new technique allowed us to sequence all the exons of these two genes.

**Figure 3:**
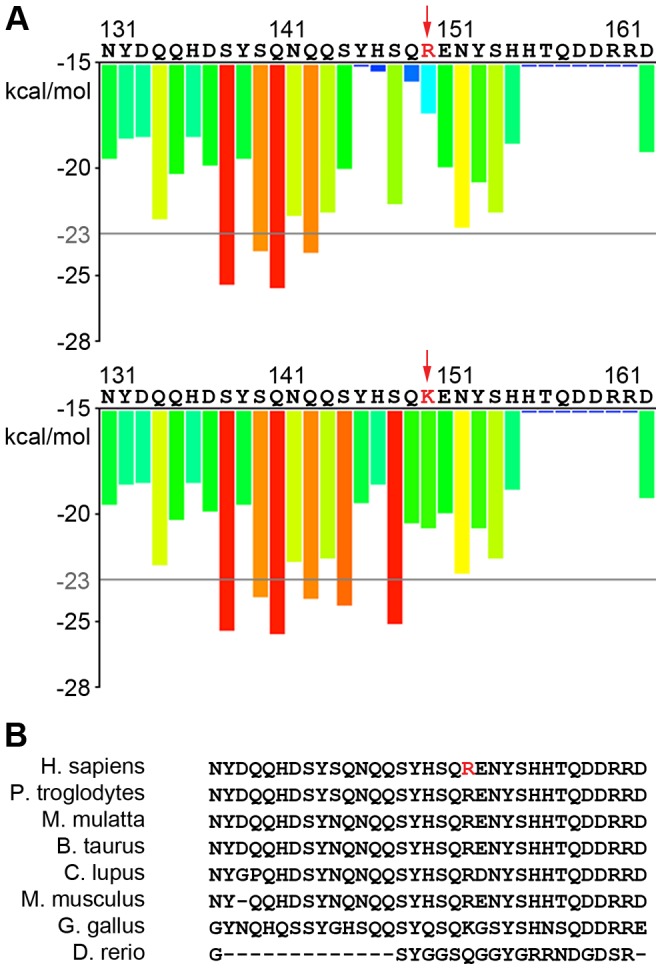
Predicted effects of new TAF15 variant on aggregation and conservation. A) ZipperDB prediction of the increase of the TAF15 p.R150K variant fibrilization propensity versus the wild type. B) TAF15 p.R150 residue is highly conserved within mammals.

We found two mutations in TAF15, one novel, p.R150K, and one rare, p.R385H. The p.R385H variant, which is present in the ESP6500 population at the same frequency as SOD1 p.D91A, 0.000538, is located in the second RGG domain and is very close to previously identified TAF15 mutations, p.M368T, p.G391E, p.R408C, and p.G473E, which are all also in the second RGG domain (Figure 1C) [7]. In contrast, p.R150K is located in the prion-like domain, which is in the N-terminal domain of the protein. Given that prion-like domains are defined by the predicted propensity of the protein sequence for fibril formation, we asked if the p.R150K variant would increase the fibrillization propensity of TAF15. Using ZipperDB, which predicts the fibrillization propensity of each 6 amino acid stretch within a protein [53]. As shown in Figure 3A, the p.R150K mutation increases the predicted fibrillization propensity of TAF15. Additionally, this is a highly conserved residue, suggesting that changes at this amino acid could be detrimental (Figure 3B).

We also found a rare variant in EWSR1, p.Y190F, which is also in the prion-like domain. However, when analyzed in ZipperDB, this variant does not alter the fibrillization propensity of EWSR1. Similarly to TAF15, previously identified EWSR1 variants were found in the C-terminal domain of the protein [8], while this rare variant is located in the N-terminal domain.

### Genes associated with ALS

While only about 20 genes have been conclusively linked to ALS, many more have been studied in connection to ALS. For example, as of November 2013, there are 17 studies returned in a search for ALS GWAS in the catalog of published genome-wide association studies maintained by the National Human Genome Research Institute (http://www.genome.gov/gwastudies). In these studies, approximately 70 regions were associated with ALS over the control populations. While some of these emerged in several studies (e.g. the region containing *C9ORF72*), many only appear once [18]. Several SNPs have also been linked to ALS through other means, including candidate gene approaches (e.g. [54]). Currently, many genes linked to ALS are of unknown significance in the disease, as studies often report conflicting results (e.g. [54] vs. [55]). We therefore asked of approximately 80 of these genes (associated) if we could find new variants or confirm previously identified ones in our patient population (Table S1, S2).

#### SNPs previously associated with ALS

Since we sequenced all exons of genes that were previously linked to ALS through GWAS or other association studies, we could simultaneously ask of multiple genes if we saw an association between a previously published SNP and ALS in our patient population. Although many identified SNPs reside outside of exons, we were able to analyze fourteen SNPs located within exons in ten genes (*ALAD*, *ANG*, *APEX1*, *APOE*, *HFE*, *OGG1*, *PON1*, *PON2*, *PVR* and *SOD2*) and, with the exception of *APOE*, which is discussed further below, we were unable to find an association between any of these SNPs and ALS in our cohort [54]–[86] (Table 3). We did, however, discover several rare and novel variants in these and other associated genes in our ALS samples (Tables S2, S4).

**Table 3:** SNPs previously associated with ALS.

Gene	Variant	dbSNP	In ALS	In Controls	P-Value	Reference
ALAD	p.K59N	rs1800435	26	16	0,87	[56]
ANG	p.G110G	rs11701	59	32	1	[85], [86]
APEX1	p.D148E	rs1130409	163	88	0,67	[57]–[60]
HFE	p.H63D		64	36	1	[54], [55], [69]–[72]
HFE	p.C102Y		32	15	0,63	
HFE	p.H63D homozygous		8	1	0,17	
HFE	p.H63D and p.C102Y		9	3	0,55	
HFE	p.H63D hom OR p.H63D and p.C102Y		17	4	0,11	
OGG1	p.S326C	rs1052133	71	50	0,14	[73]
PON1	p.L55M	rs854560	101	58	0,91	[74]–[78]
PON1	p.Q192R	rs662	51	22	0,28	[74]–[78], [80]
PON2	p.A148G	rs12026	32	11	0,17	[74]
PON2	p.S311C	rs7493	91	41	0,18	[74], [76]–[79]
PVR	p.A67T	rs1058402	26	17	0,62	[81]
SOD2	p.V16A	rs4880	89	57	0,33	[82]–[84]

Number of variants in ALS and control samples for fourteen SNPs in ten genes (*ALAD, ANG, APEX1, APOE, HFE, OGG1, PON1, PON2, PVR* and *SOD2*) previously linked with ALS [54]–[86].

#### APOE

Apolipoprotein E (APOE) has previously been linked to Alzheimer's disease, where the ε4 allele is a risk factor and the ε2 allele is considered protective [87]. There are three alleles of APOE – ε2, ε3, and ε4 – which are determined by two SNPs, p.C130R (rs429358) and p.R176C (rs7412). The ε3 allele is the “wild type” state and is most common in the population (p.C130/p.R176), while the ε2 (p.C130/p.R176C) and ε4 (p.C130R/p.R176) alleles are less common [87]. More recently, the presence of the ε4 allele has been linked to increased risk, faster progression or earlier age of onset in some neuromuscular diseases, while the ε2 allele is associated with a better prognosis [88]. Several studies have examined the association of the *APOE* alleles with different aspects of ALS, with varying and often conflicting results [61]–[68].

We asked of our patient cohort if we saw any association with the ε2 or ε4 allele and different ALS phenotypes. Thus far, no significant effect of the ε4 allele has been demonstrated in ALS overall [67], and we also saw no enrichment in the ε4 allele across all of the patients we sequenced versus the controls; however, we did see effects of the ε2 and ε4 alleles when we analyzed specific ALS phenotypes. The ε2 allele is generally considered to be protective and has been associated with limb onset, while the ε4 allele is detrimental and has been associated with bulbar onset, in some populations, although not in others [61], [62], [65], [67]. While we saw no association of the ε4 allele with bulbar onset ALS, we did see an association of the ε2 allele with upper limb onset (Fisher's Exact test, p = 0.032, OR = 5.22, 95% CI: 1.14–34.31, Table S8). Conversely, the ε2 allele has been associated with a later age of onset [64], which we did not observe in our population. The ε2 allele has also been associated with longer duration of disease [65], while the ε4 allele has been linked to a shorter survival time [63]; one of the Coriell panels that we sequenced consisted of patients with greater than seven years survival (Coriell plate NDPT025), so we asked if there was an enrichment of the ε2 allele in these patient samples versus an enrichment for ε4 in the other patient samples that we sequenced. We saw no association with the ε2 allele and increased duration of disease or with the ε4 allele and decreased disease length. Late-onset Alzheimer disease is more common in females than in males, and is also correlated with a higher prevalence of the ε4 allele in females with late-onset Alzheimer disease [89]–[91]. It has also been previously shown than female ε4 carriers had more severe brain lesions, especially in the quantity of amyloid plaques and neurofibrillary tangles [92]. As such, we investigated a potential gender bias in the contribution of APOE alleles to ALS. In the case of limb onset ALS, we also saw enrichment in female patients harboring the ε4 allele versus males (Fisher's Exact test, p = 0.012, OR = 4.13, 95% CI: 1.24–13.66, Table S8 and a trend towards it versus female controls (Fisher's Exact test, p = 0.082, OR = 2.35, 95% CI: 0.88–6.16, Table S8). Our results suggest that the ε2 allele is acting protectively in our population, as it is associated with limb onset ALS, which is often less severe than bulbar onset ALS. Additionally, we find that the ε4 allele is detrimental, as it is linked to an earlier age of onset in limb onset ALS. Finally, we find that the ε4 allele is more frequent in females than males with limb onset ALS.

#### Genes associated with other degenerative diseases

Given the similarities of many degenerative diseases (e.g. age-related, presents with accumulation of misfolded proteins), it is not unreasonable to suspect that causative mutations in one degenerative disease might play a role in another. In light of this, several genes linked to other macular, muscular and neurodegenerative diseases have been studied in the context of ALS, including *APOE* (Alzheimer's Disease, AD), *CST3* (age-related Macular Degeneration, MD), *OPTN* (Open Angle Glaucoma, OAG), *GRN* (Frontotemporal dementia, FTD), and *PSEN1* (AD) [61], [63]–[65], [67], [68], [93]–[104]. While we did not see a significant association between any of these disease genes and ALS, we did identify variants previously linked to other neurodegenerative diseases in our patient and control populations (Table S6). We also identified novel and rare variants in some of these genes (Table S2, S4); for example we found two novel variants in PSEN1: p.W203C, which is close to the known AD variant p.G206A, and p.I249L which is near the known AD variants p.A246G, p.L250S, and p.Y256S [105]–[108]. The novel variants that we identified in these genes may serve as a resource in the study of other degenerative diseases as well as ALS (Table S2).

#### Frequencies of genes associated with ALS

Several of these genes were also recently sequenced in an Irish population [35], allowing us to compare the frequencies with which these genes were present in the two populations (Table 2). In the Irish population, the gene in which the authors found the most variants was *SPG11* [35]. Mutations in *SPG11* are most frequently associated with autosomal recessive spastic paraplegia with thin corpus callosum, which presents with progressive weakness and spasticity of the lower limbs caused by degeneration of corticospinal neurons [109]; however, recently mutations were found in *SPG11* in a family with recessive juvenile ALS [110]. We also found many variants in *SPG11*; there were novel or rare *SPG11* variants in 4.13% of our patient population (n = 10). However, we also found *SPG11* variants in 3.10% of our control population (n = 4), suggesting that the frequency with which *SPG11* variants are encountered is perhaps due more to the large size of the gene than to a link with ALS in our population, although we cannot rule either possibility out. It is worth noting that one of our patient samples contained two novel variants in SPG11, a stopgain (p.Q541X) and a nonsynonymous change (p.F2063L). As these two positions are far apart, we cannot tell from our sequencing data whether the mutations are in *cis* or *trans*. This stopgain in SPG11 is one of only 5 novel or rare stopgains identified in our patient samples, compared to none in our control samples.

### Enrichment of deleterious alleles in ALS-related genes in cases vs. controls

We looked for enrichment of deleterious rare or novel alleles in known and associated genes using a modified version of a previously described method for allele binning [28]. We focused on known and associated genes because these genes have a high prior for involvement in ALS, and this method has been previously shown to work specifically using genes with prior biological evidence. This method incorporates Polyphen-2 scores and thus downweights any variation that is not predicted to be deleterious to the protein. Using this method, we found a significant difference in the distribution of deleterious variation between cases and controls (Fisher's Exact test, p = 0.019, OR = 1.93, 95% CI: 1.11–3.51, Table S8).

### Candidates from trio analysis

We previously attempted to identify new ALS disease genes in sALS cases by studying trios, which are made up of two unaffected parents and an affected proband – the idea being that the proband will have a *de novo* mutation that is not present in either parent [15]. This approach has previously been used to great effect to study the genetics of autism, intellectual disability, and schizophrenia [111]–[117] and when applied to ALS trios revealed several *de novo*, novel variants [15]. We therefore asked if we could identify further variants in these ALS candidate genes from the trio analysis in a larger data set, so we sequenced several of these genes as part of our HaloPlex analysis.

One of the variants identified in the ALS trios study was a nonsense mutation that removed the last nine amino acids of a protein called SS18L1/CREST. SS18L1 is a member of the nBAF complex and is thus involved in chromatin remodeling and it has also been implicated in neurite outgrowth [15], [118]–[121]. Additionally, a second novel missense mutation was found in one member of a family with ALS, p.I123M [15]. More recently two new SS18L1 variants were identified in a cohort of 87 fALS patients: one novel missense mutation, p.A264T, and one deletion, p.G222_S224del [122]. In this new patient cohort we found a novel variant in SS18L1 at p.G168V and a rare variant at p.G151S; no novel or rare variants were found in the control samples (Tables S2–S5).

In addition to *SS18L1*, several other genes involved in chromatin regulation were also enriched in the dataset, including *EHMT1*, *FOXA1*, *HDAC10*, and *SRCAP*, which was one of the genes with the most rare or novel variants in our HaloPlex dataset (Table S2) [15]. We identified 10 novel or rare variants in *SRCAP* in patients versus only 2 in controls (Tables S1–S5, Figure 2). *SRCAP* has a very low RVIS (Residual Variance Intolerence Score) at −4.14 (Table S1). RVIS scores are a measure of genic intolerance to functional variation and low RVIS scores have been shown to be a good predictor of whether or not a variant is likely to be disease causing, as a gene with very high genic intolerance (a low RVIS score) would be less likely to tolerate random variation, and thus variations within that gene are more likely to be deleterious [123]. However, it is worth noting that *SOD1*, in which virtually any mutation will lead to ALS, has a relatively high RVIS score of −0.08.

A *de novo* frameshift was also identified in *SRCAP* in the trio study, which results in truncation of the protein, similar to *SSI8L1* [15]. Also similar to SS18L1, SRCAP is a CBP-interacting transcriptional co-activator [124]. Heterozygous mutations in *SRCAP* have been extensively linked to Floating-Harbor syndrome, which is characterized by low birth weight, short stature, skeletal anomalies, and intellectual disability [125]. If *SRCAP* is indeed an ALS disease gene, this further underscores the potential link between neurodevelopmental and neurodegenerative diseases [15].

As for the other three chromatin remodeling genes that came out of the trio study, we found 5 novel and rare variants in *EHTM1*, and 1 novel variant each in *FOXA1* and *HDAC10* (Table S1–S5) [15].

### RNA binding proteins and ALS

The central role that RNA binding proteins play in ALS has been emerging over the past few years, as more and more RNA binding proteins surface as ALS disease genes [1], [126], [127]. This list includes *ANG*, *ATXN2*, *FUS*, *TARDPB*, *TAF15*, *EWSR1*, *SETX*, and more recently *hnRNPA2/B1* and *hnRNPA1* [1], [9]. Several of these genes contain both RNA Recognition Motif domains (RRM), a small ∼80 amino acids domain that binds single-stranded RNA [128], and a prion-like domain [7], [126], [127]. We wondered whether there might be an increased load of rare or novel variants in other proteins that had RRM and prion-like domains in ALS cases. In this experiment, we sequenced 30 RRM genes with the highest prion domain scores or the highest toxicity scores that were identified in our previously published yeast assay [7], [8]. While we did not find any enrichment in variants in this gene set, we did find a number of rare and novel variants in these genes, suggesting that sequencing a larger set of genes in a bigger sample set in the future would be beneficial (Table S2, S4). In addition to the rare and novel variants in *TAF15* and *EWSR1* discussed above, we also found 17 novel and rare variants in genes including *RBM33*, *CELF4*, and *SFPQ* (Table S1, S2, S4). Further specific examples are discussed below.

#### hnRNP genes and ALS

Recently, a family of RNA binding proteins called hnRNPs has been linked to neurodegenerative and neuromuscular diseases, including to ALS. Mutations in *hnRNPA1* and *hnRNPA2/B1* were recently shown to cause familial inclusion body myopathy with frontotemporal dementia, Paget's disease of bone, and ALS, and mutations in *hnRNPA1* were also found in cases of sALS [9]. Other members of the hnRNP family were recently associated with neuromuscular diseases; hnRNPA3 was found in cytoplasmic inclusions in ALS and FTLD cases caused by a hexanucleotide expansion in *C9ORF72* [129] and HNRPDL was identified as a cause of limb-girdle muscular dystrophy 1G [13]. We sequenced members of the hnRNP family and identified several rare and novel variants. We found relatively rare variants in in hnRNPA0, p.G187S, which was not present in any of the control cases (rs201091840; ESP6500 0.000625) and in hnRNPAB, p.G254D (rs141539534; ESP6500 0.000769). Because we used the *SOD1* p.D91A variant frequency of 0.000538 as a cut off these variants were not included in our rare variant tables or analyses. We also found novel variants in patients in hnRNPA1 (p.G192E) and hnRNPD (p.Y275H) (Table S2).

#### Stress granules and ALS

As the role of RNA binding proteins in ALS has become more and more apparent, researchers have begun to ask what role these RNA binding proteins might play under physiological conditions and in disease. Stress granules are RNA protein granules that form transiently in cells in times of stress; mRNAs and associated RNA binding proteins are sequestered into stress granules, thus preventing their translation and allowing the cell to focus its resources on surviving the stress and only translating mRNAs essential for survival [130]. Several recent studies have linked ALS and stress granules; stress granule proteins have been found colocalized with protein inclusions in patient samples and in models of the disease and several ALS disease proteins are also stress granule proteins, including TDP-43, FUS, TAF15, EWSR1, ANG, PFN1 and ATXN2 [126], [131]–[134]. A subset of the RNA binding genes that we sequenced in our study are also stress granule genes and we asked if we could identify any trends or novel variants in our patient samples versus controls. We found 10 novel or rare variants in stress granule genes in patients (4.13%) and only 1 in controls (0.78%) (Fisher's Exact test, p = 0.106, OR = 5.45, 95% CI: 0.77–117.92, Tables S8 and S9). One of these was a novel variant in ANG, p.A24T; point mutations scattered throughout this small gene cause both sporadic and familial ALS [86], [135]–[139].

It is also worth commenting on what results we did not find. Mutations in *TIA1* were recently linked to Welander distal myopathy [10], [11]; this fact combined with the role of *TIA1* as a core stress granule gene lead us to hypothesize that we might also find mutations in *TIA1* in sALS cases. However, we found no rare or novel variants of *TIA1* in our patient set, suggesting at least preliminarily that mutations in *TIA1* do not contribute to sALS.

## Discussion

We used a candidate gene approach to discover new mutations in biologically relevant ALS genes using sporadic ALS cases. Additionally, we used a large array of tools to study newly identified variants and assess relevance to disease. We used functional studies, when possible, to compare newly identified variants with other ALS variants. We also used bioinformatics tools to assess variant pathogenicity and combined it with a rare variant binning approach to demonstrate that cases have a higher burden of deleterious mutations than controls. This supports the idea that novel or rare mutations in a set of key genes may contribute to sporadic ALS.

The abundance and relative affordability of next generation sequencing techniques has launched a new era in the study of human disease. Suddenly, the capacity to discover the causative mutation behind disease seems almost at one's fingertips. This approach is more straightforward in homogenous diseases, or in cases where large families are available. However, for a heterogeneous disease like ALS, where the majority of the cases are sporadic, more creative approaches are required. We took advantage of the HaloPlex target capture system (Agilent) to rapidly sequence a set of 169 candidate genes in 242 patients and 129 controls to ask if we could (1) identify new mutations in known ALS disease genes, (2) find further evidence that genes previously associated with ALS are indeed causative, (3) find new mutations in candidates generated from our previously performed study with ALS trios and finally, (4) ask if we could find variants in RNA binding proteins.

This candidate gene approach has both benefits and drawbacks. By focusing on a list of genes, those genes could be sequenced more rapidly and cost effectively than whole exome or genome sequencing. However, there is an inherent bias in this method since we are only testing the genes we chose to sequence, and could very well be missing important variants in genes outside of our gene set. Thus, even when using a whole exome or genome sequencing approach with a limited number of patients, the first pass analysis focuses on biologically relevant candidate genes. Therefore, a candidate gene based panel may be a more cost effective way of assessing sporadic disease in a particular population.

For example, many neuromuscular diseases have similar clinical manifestations, and can be challenging to diagnose precisely in their early stages. A panel of ∼500 known causative genes can easily be created as a "neuromuscular diagnostic kit" and be sequenced looking for known or novel variants. This approach would be faster and cheaper than the current practice of iteratively performing dozens of traditional Sanger sequencing tests and would considerably help to streamline clinical diagnostic processes. Alternatively, this technique could be used as it was in this study, to quickly probe a large set of potential disease genes, for which there is not conclusive evidence of their linkage to a disease.

Using the novel approach of sequencing many candidate genes in many patients and controls using the HaloPlex method has yielded a plethora of genetic information. Viewed as a whole, we found a statistically significant enrichment of deleterious novel and rare variants in patients versus controls, suggesting that deleterious variation across a set of biologically relevant genes may be responsible for sporadic ALS. Our method, which combined polyphen-2 scores and binned variation, combined with functional studies and additional bioinformatics approaches allowed us to have statistical power to identify a difference between two groups. A common problem for assessing rare variation is the lack of statistical power – here we demonstrated an example of an allele binning strategy as a means of showing the importance of a biologically relevant set of genes.

When we looked closer at our dataset, we found several interesting variants in each of the four categories of genes that we tested (known ALS genes, genes associated with ALS, genes from a previous trio analysis and genes with RRM). For example, we provide further evidence that point mutations *ALS2* and *SETX* may cause adult onset ALS in addition to their tradition role as juvenile ALS disease genes [35], [39]–[42], [44]. We found a novel variant in FUS (Y526C), which in a functional study caused mislocalization of the protein from the nucleus to the cytoplasm, similar to other disease-causing FUS variants (Figure 2). When we analyzed genes associated with ALS, we found that in our patient population the ε2 allele of *APOE* is associated with limb onset ALS, while the ε4 allele is associated with an earlier age of onset in limb onset ALS, and is also more frequent in female limb onset ALS patients. When we sequenced the genes uncovered in our previously published trio analysis [15], we found many novel and rare variants in *SRCAP*, suggesting that it merits further study as a potential ALS disease gene. Finally, we identified many novel and rare variants in genes containing RRM motifs, including in *hnRNPA1* and *hnRNPA2/B1*, which have both been recently linked to ALS [9].

In this report, we have analyzed only nonsynonymous variants, as those are the changes that we best understand the consequences of. However, a huge portion of the variants that we identified result in synonymous changes; thus there is a large untapped part of our dataset that may turn out to be relevant to disease. This will be an area for future exploration and data analysis, as our understanding of the effects of synonymous variation grows. But even for nonsynonymous changes, assessing pathogenicity for a given variant can be very challenging. For known genes, were their role in ALS is already fairly well characterized (e.g., *SOD1* and *FUS*) it is reasonable to design functional studies to verify that newly identified variants behave similarly to the ones previously linked with ALS. This is the approach we took to assess pathogenicity of the novel Y526C FUS variant (Figure 2). Since this variant is located within a conserved NLS and nearby other pathogenic mutations (e.g., P525L), we were able to test the effect on FUS nuclear localization and demonstrated that the mutation caused redistribution to the cytoplasm (Figure 2), in support of the pathogenicity of the variant. For other candidate genes from our study, like *SPG11*, *SRCAP*, *AIM1L*, it is less feasible to design studies to assess the impact of variants of different classes for function, when much less is known about the normal functions of these genes. Some of these candidate genes are involved in other diseases, like *SPG11* for example, which is also involved in Hereditary Spastic Paraplegia [109]. Are the functional studies used to test for these diseases relevant to ALS? *SRCAP*, which encodes a chromatin-remodeling factor [140], implicates potential effects on chromatin remodeling. However, are alterations in the chromatin-remodeling activity of SRCAP responsible for ALS, or is it another still unknown function? Finally, for genes like *AIM1L* (absent in melanoma 1-like), where almost nothing is known about the function of the gene product, designing informative functional studies to assess the effect of variants is not feasible. Even if a variant does functionally impact the protein it still may not be pathogenic. These issues make it a challenge to distinguish true disease-causing variants from benign ones. Clearly, new rigorous approaches will be required to help make the results of sequencing studies like this one clinically informative [141].

Familial ALS appears to be a much more homogenous disease than sporadic ALS, as approximately 20 known genes can explain about 60% of cases [1]. However, much fewer than 60% of sporadic cases have a definitive genetic cause, with mutations in known disease causing genes appearing at much lower frequencies than in fALS [1]. For example, one study found that expansions in *C9ORF72* were present in ∼40% of fALS cases, but only in ∼7% of sALS cases [142]. This suggests that the genetic causes of sporadic ALS are likely to be many and varied, that the well known ALS disease genes can only explain so much, and that broad techniques will be necessary to identify causative mutations in sALS. It is the hope as well that, just as mutations in genes discovered in fALS have been informative for sALS, the reverse will be true. Additionally, as the lines continue to blur between various neurodegenerative diseases, causative mutations found in sALS may also be relevant to other neurodegenerative diseases, such as frontotemporal dementia, Alzheimer's disease, and Parkinson's disease.

As a proof of principle study, we believe that our results prove the utility of combining sporadic ALS samples with the power of next generation sequencing techniques and that future studies will yield useful genetic information for the study of ALS in general. By taking advantage of this untapped resource, there is a whole new set of samples available for study. An oligogenic hypothesis in which sporadic ALS appears and disappears randomly as the multiple mutations necessary for disease arise in some individuals due to the random shuffling of alleles from one generation to the next is emerging, and will perhaps be best studied using next generation sequencing techniques [47]. We believe that future studies using next generation sequencing techniques on large numbers of sALS patient samples will be fruitful for further understanding the genetics of ALS. As our knowledge of ALS grows, it seems more to be a field of icebergs connected under the surface, rather than just one, underscoring the importance of continued genetic studies of ALS.

## Methods

### Patient phenotypes

We sequenced three ALS panels and two control panels from the Coriell collection of North American Caucasian DNA samples (http://www.ccr.coriell.org). NDPT025 contained samples from ALS patients that survived for 7 years or longer, NDPT026 contained patient samples from ALS patients with bulbar onset, and NDPT103 contained patient samples from ALS patients with upper-limb onset. Only patients without a family history of ALS were included in these panels and all patients met the EI Escorial criteria for definite, probable or possible ALS. Of the 242 patient samples that we sequenced, 111 were females and 131 were male and the age of onset ranged from 44 to 82 years of age, with an average age of onset of 60. More detailed information about each patient is publicly available and can be found through Coriell by searching for the plate or sample number provided on Coriell website. We sequenced 129 samples from two panels of age-matched controls, NDPT084 and NDPT099, with age at sampling from 55–88 and no family history of neurological disease, of which 69 samples were from women and 60 from men.

### Library design and preparation

Using the Agilent SureDesign online tool (https://earray.chem.agilent.com/suredesign/), a HaloPlex custom kit (Agilent, Santa Clara, CA, https://www.agilent.com) was designed to include all exons of target genes previously linked with ALS and listed in ALSoD [143] (http://alsod.iop.kcl.ac.uk/) (associated and known ALS categories), candidates genes from our previous study using ALS Trios [15] (trio category), and finally RRM genes harboring a high prion score domain or with a high toxicity score [7], [8] (RRM category) (Table S1). Five indexed paired-end Illumina sequencing libraries [144] were generated according to the HaloPlex HaloPlex manufacturer's protocol, one DNA library per 96-well plate.

### Sequencing

Sequencing was performed with 150 bp paired-end reads on an Illumina MiSeq machine. About 30% of the SNPs were Sanger sequenced. First, we Sanger sequenced many SNPs to select a highly false positive discriminating bioinformatics filter set, dropping from 20% to less than 1% false positive rate. We also selected some genes depending their significance (SPG11, SRCAP, AIM1L, FUS, TAF15, APOE ε status) to be Sanger sequenced to confirm the variants identified using the MiSeq. Finally the few SNPs identified with a coverage between 5 and 10x where also verified by Sanger sequencing.

### Bioinformatics

FastQ reads were aligned to the human reference genome (UCSC hg19, GRCh37, Feb. 2009 release) using bowtie2 [145] and SAMtools [146]. We applied GATK [147], [148] base quality score recalibration, indel realignment, duplicate removal, and performed coverage calculations, SNP and INDEL discovery and genotyping across each sample using optimized custom hard filtering parameters or variant quality score recalibration (raw variants were filtered using the following parameters: DP<5.0, QUAL<30.0, QD<2.0, FS>50.0, HaplotypeScore>13.0, MQ<30.0, MQRankSum <−12.5, ReadPosRankSum <−8.0).Variants were filtered against dbSNPv137, 1000 genomes and ESP 6500 databases and were then annotated using ANNOVAR [149].

### Statistics

Fisher's Exact tests were performed to determine if there were any enrichments among genes or categories of genes using the exact2×2 package [150] in the statistical programming tool R (version 3.1.1).

### Allele binning strategy

We used a modified version of the method previously described [28]. However, since rare and novel variants are not in linkage disequilibrium (LD) with one another, we did not weight each variant by LD but by polyphen-2 score. For testing the distribution of deleterious variation score between cases and controls, we counted the number of individuals with a score ≥0.85 (the cutoff for “probably damaging” in polyphen-2 [151], Table S8).

### Identification of novel and rare variants

Novel variants were determined to be those that were not present in either dbSNPv137, ESP6500 or 1000 genomes databases.

Rare variants were determined to be those that were present at a frequency of less than or equal to 0.000538 in the ESP6500 database. This value was chosen as it is the frequency which the ALS causative known SOD1 variant, p.D91A, is present in this database; we identified this variant in two patients. This subset also included variants which were present in dbSNPv137 at a very low or with no frequency listed, but were not present in ESP6500 and either not present or present at less than 0.01 in the 1000 genomes database.

Score and phenotypic impact predictions, when available, are provided for each variant using various algorithms. For each, a higher score indicates that a variant is predicted to be more deleterious. Algorithms used are PhyloP [152] (C: conserved, N: not conserved); SIFT [153] (D: deleterious, T: tolerated); PolyPhen2 [154] (D: probably damaging, P: possibly damaging, B: benign); MutationTaster [155] (A: disease causing automatic, D: disease causing, N: polymorphism, P: polymorphism automatic).

### FUS functional studies in mammalian cells

N2A cells were plated on top of cover slides in 24-well format and transfected with Lipofectamine 2000 (Invitrogen) according to the manufacturer's instructions. Cells were fixed in 4% PFA 24 h post-transfection and immunostained using primary anti-V5 mouse monoclonal antibody (R960-25 Invitrogen) and secondary Alexa Fluor 488 goat anti-mouse IgG antibody (A-11001 Invitrogen). Cells were mounted on microscope slides using ProLong Diamond antifade with DAPI (P36962 Invitrogen) and imaged using a Leica DMI600B microscope.

## Supporting Information

Table S1 Candidate genes sequenced and number of variants found. Genes with a poor coverage in the capture library, leading to few or no sequencing reads, are indicated in red. The candidate genes were sorted into four categories: known ALS disease genes (Known ALS), genes potentially associated with ALS (Associated), candidate genes from our previously published analysis of ALS trios (Trios) [15] and genes containing RNA Recognition Motifs (RRM) from our previously published yeast screen [7]. Some genes may fall into multiple categories. Genes with a negative Residual Variation Intolerance Score (RVIS) [123] have less common functional variation in the general population. RVIS is associated with a percentile score, reflecting the gene ranking among the most variation-intolerant human genes.
(XLSX)

Table S2 List of all novel variants identified in ALS cases. Variants were considered as novel when not present in the ESP6500, 1000genomes or dbSNPv137 databases. Score and phenotypic impact predictions, when available, are provided for each variant using various algorithms. For each, a higher score indicates that a variant is predicted to be more deleterious. Algorithms used are PhyloP [152] (C: conserved, N: not conserved); SIFT [153] (D: deleterious, T: tolerated); PolyPhen2 [154] (D: probably damaging, P: possibly damaging, B: benign); MutationTaster [155] (A: disease causing automatic, D: disease causing, N: polymorphism, P: polymorphism automatic).
(XLSX)

Table S3 List of all novel variants identified in controls. Variants were considered as novel when not present in the ESP6500, 1000genomes or dbSNPv137 databases. Score and phenotypic impact predictions, when available, are provided for each variant using various algorithms. For each, a higher score indicates that a variant is predicted to be more deleterious. Algorithms used are PhyloP [152] (C: conserved, N: not conserved); SIFT [153] (D: deleterious, T: tolerated); PolyPhen2 [154] (D: probably damaging, P: possibly damaging, B: benign); MutationTaster [155] (A: disease causing automatic, D: disease causing, N: polymorphism, P: polymorphism automatic).
(XLSX)

Table S4 List of all rare variants identified in ALS cases. Variants were considered as rare when they were present at very low frequency (≤0.000538) in the ESP6500, 1000genomes or dbSNPv137 databases. Score and phenotypic impact predictions, when available, are provided for each variant using various algorithms. For each, a higher score indicates that a variant is predicted to be more deleterious. Algorithms used are SIFT [153] (D: deleterious, T: tolerated); PolyPhen2 [154] (D: probably damaging, P: possibly damaging, B: benign); MutationTaster [155] (A: disease causing automatic, D: disease causing, N: polymorphism, P: polymorphism automatic).
(XLSX)

Table S5 List of all rare variants identified in control cases. Variants were considered as rare when they were present at very low frequency (≤0.000538) in the ESP6500, 1000genomes or dbSNPv137 databases. Score and phenotypic impact predictions, when available, are provided for each variant using various algorithms. For each, a higher score indicates that a variant is predicted to be more deleterious. Algorithms used are SIFT [153] (D: deleterious, T: tolerated); PolyPhen2 [154] (D: probably damaging, P: possibly damaging, B: benign); MutationTaster [155] (A: disease causing automatic, D: disease causing, N: polymorphism, P: polymorphism automatic).
(XLSX)

Table S6 List of known neuronal diseases variants found our cohort. Variants were considered as known to be linked with a disease when present in the ClinVar database (https://www.ncbi.nlm.nih.gov/clinvar/). We found variants that have been previously identified to be causative of Alzheimer's Disease (AD), age-related Macular Degeneration (MD), Open Angle Glaucoma (OAG) and ALS in both controls or ALS samples. We also listed any gene carrying a novel variant identified in this study for the same sample.
(XLSX)

Table S7 List of novels variants in known ALS disease genes. For each gene previously identified as being a known ALS disease gene, we listed any novel variant found in this study. We also listed any nearby variant known to be pathogenic, such as those causative of ALS, Spinocerebellar Ataxia (SCA) or Perry Syndrome (PS).
(XLSX)

Table S8 Contingency tables for Fisher's Exact tests. Fisher's Exact tests were performed on using the exact2×2 package in the statistical programming tool R using the data included in these contingency tables.
(XLSX)

Table S9 List of novel or rare variants in stress granule genes. Variants were considered as novel when not present in the ESP6500, 1000genomes or dbSNPv137 databases and as rare when present at very low frequency (≤0.000538). When analyzed in ZipperDB, fibrillization propensity was increased for two mutations and is reported here.
(XLSX)
